# Protective Effect of the Fruit Hull of *Gleditsia sinensis* on LPS-Induced Acute Lung Injury Is Associated with Nrf2 Activation

**DOI:** 10.1155/2012/974713

**Published:** 2012-03-14

**Authors:** Jun-Young Choi, Min Jung Kwun, Kyun Ha Kim, Ji Hyo Lyu, Chang Woo Han, Han-Sol Jeong, Ki-Tae Ha, Hee-Jae Jung, Beom-Joon Lee, Ruxana T. Sadikot, John W. Christman, Sung-Ki Jung, Myungsoo Joo

**Affiliations:** ^1^Department of Korean Medical Science, Korean Medicine Hospital, School of Korean Medicine, Pusan National University, Yangsan 626-870, Republic of Korea; ^2^Department of Internal Medicine, Korean Medicine Hospital, School of Korean Medicine, Pusan National University, Yangsan 626-870, Republic of Korea; ^3^Division of Allergy, Immune and Respiratory System, Department of Internal Medicine, College of Oriental Medicine, Kyung Hee University, Seoul 130-701, Republic of Korea; ^4^Division of Applied Medicine, School of Korean Medicine, Pusan National University, Yangsan 626-870, Republic of Korea; ^5^Department of Internal Medicine, Kangnam Korean Hospital, Kyung Hee University, Seoul 135-501, Republic of Korea; ^6^Section of Pulmonary, Critical Care and Sleep Medicine, University of Illinois and Jesse Brown Veterans Affairs Medical Center, Chicago, IL 60612, USA; ^7^Division of Allergy, Pulmonary and Critical Care Medicine, Vanderbilt University Medical Center, Nashville, TN 37232-2650, USA

## Abstract

The fruit hull of *Gleditsia sinensis* (FGS) has been prescribed as a traditional eastern Asian medicinal remedy for the treatment of various respiratory diseases, but the efficacy and underlying mechanisms remain poorly characterized. Here, we explored a potential usage of FGS for the treatment of acute lung injury (ALI), a highly fatal inflammatory lung disease that urgently needs effective therapeutics, and investigated a mechanism for the anti-inflammatory activity of FGS. Pretreatment of C57BL/6 mice with FGS significantly attenuated LPS-induced neutrophilic lung inflammation compared to sham-treated, inflamed mice. Reporter assays, semiquantitative RT-PCR, and Western blot analyses show that while not affecting NF-*κ*B, FGS activated Nrf2 and expressed Nrf2-regulated genes including GCLC, NQO-1, and HO-1 in RAW 264.7 cells. Furthermore, pretreatment of mice with FGS enhanced the expression of GCLC and HO-1 but suppressed that of proinflammatory cytokines in including TNF-*α* and IL-1*β* in the inflamed lungs. These results suggest that FGS effectively suppresses neutrophilic lung inflammation, which can be associated with, at least in part, FGS-activating anti-inflammatory factor Nrf2. Our results suggest that FGS can be developed as a therapeutic option for the treatment of ALI.

## 1. Introduction

Acute lung injury (ALI) and acute respiratory distress syndrome (ARDS) are severe inflammatory diseases characterized by cellular and tissue injury, lung compliance abnormality, and gas change impairment, which frequently lead to fatal respiratory failure within hours [1, 2]. Pulmonary or extrapulmonary insults, such as pneumonia, aspiration, trauma, and sepsis, cause ALI/ARDS [3], and sepsis is the most common initiator of ALI/ARDS [4]. In bacterial sepsis, lipopolysaccharide (LPS), a major component of Gram-negative bacterial cell wall, plays a key role in inducing inflammation because it stimulates the production of proinflammatory cytokines including interleukin (IL)-8, causing the infiltration of neutrophils into the lungs of ALI patients [5, 6]. Therefore, suppressing LPS-induced inflammation has been a primary target in pharmacologic treatment of ALI/ARDS patients. 

LPS binds to its receptor, Toll-like receptor 4 (TLR4), to activate a key proinflammatory transcription factor NF-*κ*B that induces expressions of various proinflammatory cytokines and chemokines such as TNF-*α*, IL-1*β*, and MIP-1*α* [7]. On the premise that blocking NF-*κ*B activity and production of proinflammatory cytokines suppresses inflammation, numerous clinical trials have been carried out, but shown no significant effect on ALI/ARDS. Current pharmacological therapies for ALI/ARDS include inhalation of nitric oxide (NO) to relieve pulmonary hypertension and administration of corticosteroids for ARDS-related pulmonary fibrosis [8, 9]. These treatments, however, lack firm support by clinical evidence and show severe adverse effects such as toxicity caused by reactive free radicals generated from NO and prolonged neuromuscular weakness, deregulation of glucose metabolism, and sepsis caused by systemic corticosteroid administration [1, 10]. Given that the only proven treatment to improve survival is mechanical ventilation with a lung protective strategy [11], it is imperative to develop effective therapeutics for ALI/ARDS.

In traditional eastern Asian medicine, the fruit hull of *Gleditsia sinensis* (FGS) LAM (Leguminosae) has long been used to treat various respiratory symptoms such as dyspnea, orthopnea, cough with phlegm, and sore throat. In addition, it has been administered externally for the treatment of subcutaneous pyogenic infections [12]. Therefore, we postulate that the therapeutic effect of FGS is attributed to a potent anti-inflammatory activity in its constituents. In this study, we tested this possibility by using ALI/ARDS animal model. Since the underlying mechanisms for the efficacy of the remedy are largely unknown, we investigated possible mechanisms by which FGS suppresses inflammation by using macrophage cell lines. Our results show that FGS is capable of suppressing neutrophilic lung inflammation in LPS-induced ALI mouse model, which is associated with, at least in part, activation of NF-E2-related factor 2 (Nrf2), an anti-inflammatory transcription factor that plays a key role in ameliorating acute lung injury [13]. 

## 2. Material and Methods

### 2.1. Preparation of the Water Extract of *G. sinensis* Fruit Hull

The fruits of *G. sinensis *were purchased from Kwang-Myoung-Dang herb store (Pusan, Republic of Korea) and authenticated by Professor C. W. Han at the School of Korean Medicine, Pusan National University, Yangsan, Republic of Korea. A voucher specimen (number: pnukh001) is kept in the School of Korean Medicine, Pusan National University. A decoction was obtained by boiling 300 g of the fruit hulls of *G. sinensis* in distilled water for 2 hours followed by filtration through 0.45 *μ*m filter. The resultant decoction underwent a freeze-drying process to yield 60 g of powder. Appropriate amount of the powder was dissolved into phosphate-buffered saline (PBS) prior to experiment.

### 2.2. Reagents and Antibodies

3-(4,5-Dimethylthiazol-2-yl)-2,5-diphenyltetrazolium bromide (MTT), Sulforaphane, and Gram-negative *Escherichia coli* LPS (serotype 055:B5) for animal study were purchased from Sigma Chemical Co. (St. Louis, MO, USA). TLR4-specific *E. coli* LPS was purchased from Alexis Biochemical (San Diego, CA, USA). All antibodies used in this study were from Santa Cruz Biotechnology (Santa Cruz, CA, USA).

### 2.3. Animals and ALI/ARDS Model

Male C57BL/6 mice, inbred in a specific pathogen-free (SPF) facility, were purchased from the Samtaco Bio Korea, Ltd. (Osan, Korea). Animals were housed in certified, standard laboratory cages, and fed with food and water *ad libitum* prior to experiment. All experimental procedures followed the Guidelines for the Care and Use of Laboratory Animals of the NIH of Korea, and all the experiments were approved by the Institutional Animal Care and Use Committee of Pusan National University, Pusan, Republic of Korea.

Prior to LPS administration, mice were fed once with either 3.3 or 13.3 mg of FGS per kilogram of a mouse for 14 days, the amount of which was same as, or 4 times higher, respectively, than that of FGS prescribed to patients in Korean medicine clinic. A single dose of FGS was in 250 *μ*L of ddH_2_O, and feeding did not cause any adverse effect on mice. At day 15, mice were anesthetized by Zoletil (Virbac) and received a single dose of 10 mg LPS/kg body weight intranasally while control mice received sterile saline. At 24 h after the administration of LPS, mice were euthanized by CO_2_ gas. The trachea was exposed through midline incision and cannulated with a sterile 24-gauge intravascular catheter. Bilateral bronchoalveolar lavage (BAL) was performed by two consecutive instillations of 1.0 mL of PBS. Total cell numbers in BAL fluid were counted with hemocytometer. Then, the cells in BAL fluid were precipitated by a cytospin and stained for macrophages, lymphocytes, or neutrophils with Hemacolor (Merck, Darmstadt, Germany). Three hundred cells in total were counted, and one hundred cells in each microscopic field were scored. The mean numbers of the cells per field are shown.

For the analysis of lung tissue, mice were perfused with saline and the whole lung was inflated with fixatives. After paraffin embedding, 5 *μ*m sections were cut and placed on charged slides and stained with hematoxylin and eosin (H&E) staining method. Three separate H&E-stained sections were evaluated in 200x microscopic magnifications per mouse.

### 2.4. Cell Culture

RAW 264.7 cells (American Type Culture Collection, Rockville, MD, USA) were cultured in Dulbecco's Modified Eagle's Medium (DMEM) containing L-glutamine (200 mg/L) (Invitrogen; Carlsbad, CA, USA) supplemented with 10% (v/v) heat-inactivated fetal bovine serum (FBS) and 100 U/mL penicillin and 100 *μ*g/mL streptomycin (Invitrogen) and maintained in a humidified incubator at 37°C and 5% CO_2_ prior to experiment.

### 2.5. Microculture Tetrazolium (MTT) Assay

The cytotoxicity caused by FGS was assessed with MTT-based colorimetric assay (Skehan, 1998). In brief, after Griess reaction, MTT solution (2.0 mg/mL) was added to each well of cells cultured in a 96-well plate. At 4 h after incubation at 37°C in a CO_2_ cell culture incubator, the supernatants were removed, and formazan crystals formed in viable cells were measured at 540 nm with a microplate reader. The percentage of living cells was calculated against untreated cells.

### 2.6. Western Blot Analysis

Total cell extract of 5 × 10^6^ cells was prepared as described previously [14]. Nuclear proteins were isolated by NE-PER nuclear extraction kit and the manufacture's protocol (Thermo Scientific, IL, USA). The amounts of proteins were measured by Bradford (Bio-Rad Laboratories, Hercules, CA, USA). Equal amounts of proteins were fractionated by SDS-PAGE and then transferred to PVDF membrane (Bio-Rad Laboratories). Blots were blocked for at least 1 h with 5% nonfat dry milk prior to incubation with Nrf2, NF-*κ*B (p65), lamin A/C polyclonal antibodies at 4°C for overnight. After incubation with secondary antibodies conjugated with HRP for 1 h at room temperature, the bands of interest were revealed by chemiluminescence (SuperSignal West Femto, Thermo Scientific).

### 2.7. Measurement of Nitric Oxide (NO) Production

RAW 264.7 cells were treated with 2.5 or 5 *μ*g/mL of FGS for 16 h prior to LPS (100 ng/mL) treatment for 16 h. Produced NO was determined by measuring the stable conversion product of NO, nitrite (NO_2_^−^). Briefly, 100 *μ*L of cell culture medium was mixed with 100 *μ*L of Griess reagent (0.1% N-(1)-naphthyl-ethylenediamine, 1.0% sulfanilamide, and 2.5% phosphoric acid) in a 96-well plate and incubated at room temperature for 5 min prior to reading at 540 nm with a microplate reader. Sodium nitrite (NaNO_2_) was used for setting up a standard curve.

### 2.8. Reporter Constructs, Reporter Cell Line, and Luciferase Assay

For the measurement of Nrf2 transcriptional activity, an Nrf2 reporter cell line was created. From genomic DNA isolated by QIAmp DNA Mini Kit (Qiagen) and the instructions of the manufacture, the proximal 1kb-long promoter of a murine NQO-1 gene, where an Nrf2 binding site locates, was amplified by PCR with a pair of primers: 5′-GCTATGTGGACCA GTCTGG-3′ and 5′-GGCTCCAGATGTTGAGGGA-3′. The PCR product was verified by sequencing and subsequently cloned into pGL4.17 [*luc2*/Neo] vector (Promega). The resultant vector, NQO-1[*luc*/Neo], was stably transfected into RAW 264.7 cells, and candidate Nrf2 reporter cell lines were selected under G418 (Invitrogen). The Nrf2 reporter cell line was tested for its responsiveness to Sulforaphane, a well-documented Nrf2 activator. Luciferase activity was measured by a luciferase assay kit (Promega) and the instructions of the manufacturer and normalized by the amount of total proteins of the cell extract. 

### 2.9. Isolation of Total RNA from Cells and RT-PCR

Total RNA of tissue and cells was isolated with the QIAGEN RNeasy mini kit (Qiagen, Hilden, Germany) according to the manufacturer's instructions. Three micrograms of total RNA were reverse-transcribed by M-MLV reverse transcriptase (Promega, Madison, WI, USA), and single-stranded cDNA was amplified by PCR with a set of specific primers as follows: the forward and the reverse primers for NQO-1 were 5′-GCAGTGCTTTCCATCA CCAC-3′ and 5′-TGGAGTGTGCCCAATGCTAT-3′, respectively; the primers for HO-1 were 5′-TGAAGGAGGCCACCAAGGAGG-3′ and 5′-AGAGGTCACCCAGGTAGCGGG-3′, respectively; the primers for GCLC were 5′-CACT GCCAGAACACAGACCC-3′ and 5′-ATGGTCTGGCTGAGAAGCCT-3′, respectively; the primers for TNF-*α* were 5′-CTACTCCTCAGAGCCCCCAG-3′ and 5′-AGGCAACCTGACCACTCTCC-3′, respectively; the primers for IL-1*β* were 5′-GTGTCTTTCCCGTGGACCTT-3′ and 5′-TCGTTGCTTGGTTCTCCTTG-3′, respectively; the primers for GAPDH were 5′-GGAGCCAAAAGGGTCATCAT-3′ and 5′-GTGATGGCATGGACTGTGGT-3′, respectively. For PCR amplification, *Taq*PCRx DNA polymerase, Recombinant (Invitrogen, Carlsbad, CA, USA), and the manufacturer's protocol were used. The reaction conditions were as follows: an initial denaturation at 95°C for 5 min followed by 22–30 cycles of denaturation for 40 sec at 95°C, annealing for 40 sec at 57°C, and extension for 50 sec at 72°C with a final extension for 7 min at 72°C. Amplicons were separated in 1.2% agarose gels in 1× TBE buffer at 100 V for 30 min, stained with ethidium bromide, and visualized under UV light. GAPDH (Glyceraldehyde-3-phosphate dehydrogenase) was used as internal controls to evaluate relative expressions of NQO-1, GCLC, and HO-1. Relative expression of each gene over GAPDH was determined by densitometric analysis software ImageJ (Wayne Rasband, Research Services Branch, National Institute of Mental Health, Bethesda, Maryland, USA).

### 2.10. Measurement of Reactive Oxygen Species (ROS) Production

Intracellular ROS generation in RAW 264.7 cells was determined by carboxy-H_2_DCFDA (5-(and-6)-carboxy-2′,7′-dichlorodihydrofluorescein diacetate (Molecular Probes, Eugene, OR, USA). Briefly, after various pharmacological treatments, RAW 264.7 cells were treated with 100 *μ*M carboxy-H_2_DCFDA in cultured medium and incubated at 37°C for 30 min. After incubation, the cells were washed with PBS and then fluorescence was measured by using BD FACS Canto II (BD Biosciences, San Jose, CA, USA) at an excitation wavelength of 488 nm and an emission wavelength of 525 nm.

### 2.11. Statistical Analysis

For comparison among groups, one-way analysis of variance (ANOVA) tests with Tukey's post hoc test was used (with the assistance of InStat, Graphpad Software, Inc., San Diego, CA) (*P* values <0.05 are considered significant). All the experiment was performed at least three times independently. 

## 3. Results

### 3.1. The Water Extract of FGS Suppresses Acute Neutrophilic Lung Inflammation in an ALI/ARDS Mouse Model

**Figure 1:**
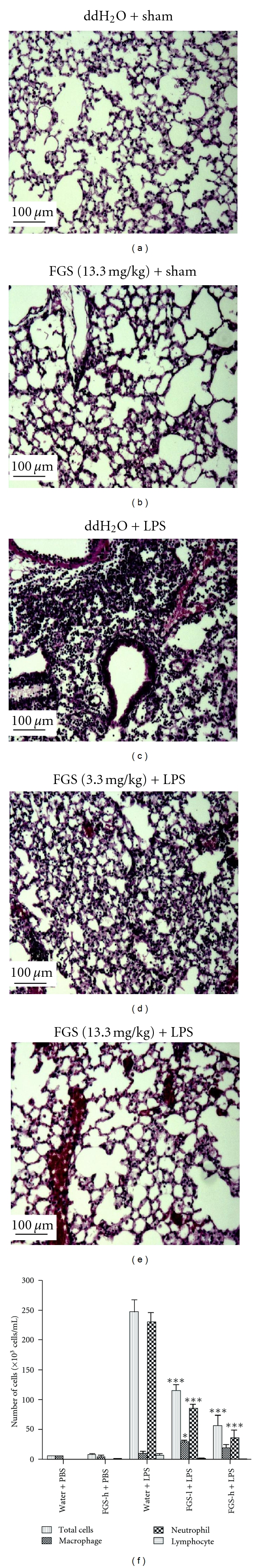
FGS suppresses acute neutrophilic lung inflammation in an ALI animal model. (a)–(e) H&E-stained lung sections of C57BL/6 mice. C57BL/6 mice, fed with either water (a) and (c) or FGS (b), (d), and (e) for 14 days, received an intranasal LPS (c), (d), and (e) or PBS (a) and (b) (*n* = 5 per group). At 24 h after LPS treatment, the lungs of mice were perfused and analyzed by histological examination (magnification × 200). LPS treatment shows significant cellular infiltrates (c), which are decreased by FGS treatments (d) and (e). Shown are representatives of at least five different areas of a lung. (f) Bronchoalveolar lavage (BAL) was performed to count infiltrates in the lung. Total cell numbers were counted by using a hemocytometer. The cells in BAL fluid were precipitated by a cytospin and differentially counted for macrophages, neutrophils, and other inflammatory cells. Data represent the mean ± SEM of three independent counting. *P < 0.05, ***P < 0.001; significantly different from group C by post ANOVA comparison with Tukey's post hoc test.

Since the aqueous extract of the fruit hull of *G. sinensis* (FGS) LAM has been prescribed to treat various respiratory diseases, we hypothesized that FGS is effective in treating inflammatory lung diseases by suppressing inflammation. To test our hypothesis, we used an ALI/ARDS mouse model, one of the hallmarks of which is neutrophilic lung inflammation. The water extract of FGS was prepared and administered orally to seven-week old C57BL/6 mice for 14 days. Mice received either 3.3 mg/kg of FGS (*n* = 10), equivalent to the dose administered to a patient per day, or 13.3 mg/kg of FGS (*n* = 10), 4 times higher than the dose for a patient. At day 15, mice were divided half, and one half received sham treatment, and the other half did an intranasal instillation of LPS (10 mg/kg) to induce acute lung inflammation. At 24 h after LPS administration, the mice were euthanized for the analysis of lung inflammation. Histological analyses of lung sections show that the mice received FGS and sham treatments kept the lung structure intact, similar to control mice treated with water and sham (Figures 1(a) and 1(b)), suggesting that FGS treatment did not provoke any inflammation. The lungs of mice received an intranasal LPS developed severe lung inflammation, as evidenced by the edematous alveolar wall thickened with severe inflammatory cell infiltration (Figure 1(c)). However, the degree of lung inflammation was significantly reduced by FGS treatments (Figures 1(d) and 1(e)). It is notable that the higher dose of FGS made the inflamed lung return close to the control level (Figure 1(e)).

We also performed differential counting of infiltrates in BAL fluid obtained from variously treated mice, showing that the numbers of total cellular infiltrates and neutrophils in the lungs induced by LPS instillation were reduced by 53.2% and 62.8%, respectively, when the mice were treated with 3.3 mg/kg of FGS (Figure 1(f)). Reduction of inflammatory cellular infiltration was more evident when the mice were treated with 13.3 mg/kg of FGS: those of cellular infiltrates and neutrophils were down by 77.6% and 84.4%, respectively (compared the 3rd group of columns to the 5th group). Together, these results demonstrate that FGS strongly suppresses acute neutrophilic lung inflammation.

### 3.2. FGS Did Not Affect NF-*κ*B Activity

**Figure 2:**
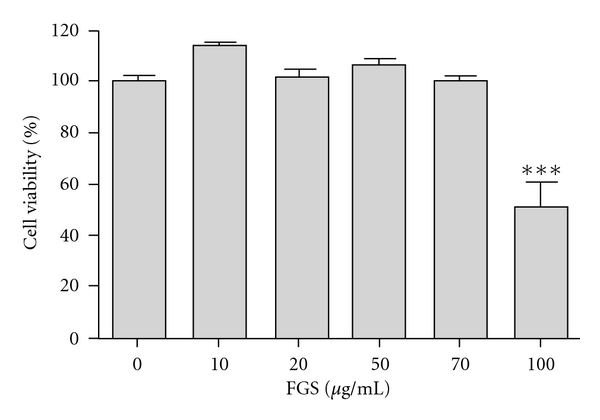
Effect of FGS on cell viability. Cytotoxic effect of FGS on RAW 264.7 cells was measured by MTT assay. The cells were treated with various amounts of FGS for 16 h prior to MTT assay. Data represent the mean ± SEM of three independent determines. ***P < 0.001, which was significantly different compared to control.

Since NF-*κ*B is a well-documented transcription factor that regulates expressions of numerous proinflammatory cytokines and NF-*κ*B activity was reported to increase in patients with acute lung injury [15], we tested the possibility that FGS exerts its anti-inflammatory function by suppressing proinflammatory NF-*κ*B activity. First, to determine an optimal dose of FGS without significant cellular toxicity, we performed MTT assays with RAW 264.7 cells, a murine macrophage cell line. The cells were treated with various amounts, from 10 to 100 *μ*g/mL, of FGS for 16 h prior to MTT assay. As shown in Figure 2, FGS did not show any significant cellular toxicity except 100 *μ*g/mL, in which a slight cytotoxicity was detected. For the study, we used least, yet effective, amounts of FGS, 2.5 and 5 *μ*g/mL.

**Figure 3:**
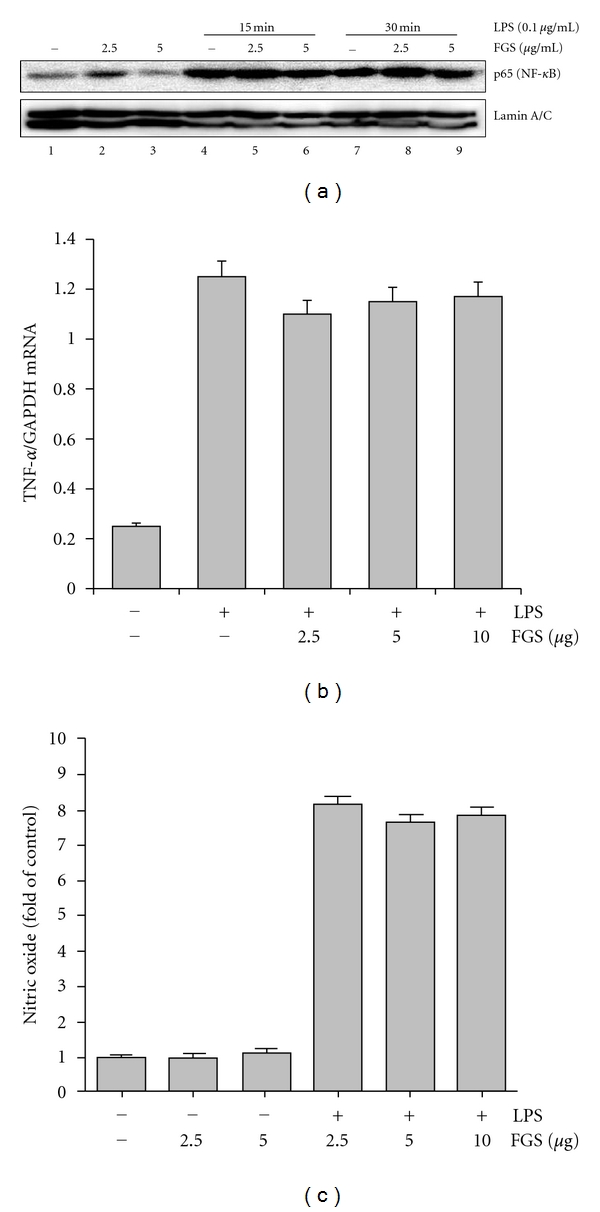
FGS does not affect NF-*κ*B activity. (a) The effect of FGS on the emergence of nuclear p65, indicative of NF-*κ*B activation, was determined by Western Blot analysis. RAW 264.7 cells, pretreated with various amounts of FGS for 16 h (lanes 2, 3, 5, 6, 8, and 9), were further treated with LPS (100 ng/mL) for 15 min and 30 min (lanes from 4 to 9). The blot was stripped and reprobed against nuclear proteins lamin A/C to ensure an equal loading. (b) RAW 264.7 cells, pretreated with 2.5, 5, or 10 *μ*g/mL of FGS for 24 h, were treated with LPS (100 ng/mL) for 16 h, and total RNA was extracted and analyzed by semiquantitative RT-PCR for TNF-*α*. The intensity of each PCR product was measured by densitometric analysis (ImageJ), and relative expression of TNF-*α* was calculated over GAPDH. (c) RAW 264.7 cells were treated similarly to (b), and NO produced by the cells was measured. Data represent the mean ± SEM of three independent experiments.

Next, we tested whether FGS affects NF-*κ*B activity. RAW 264.7 cells, pretreated with two different amounts of FGS (2.5 *μ*g/mL or 5 *μ*g/mL) for 16 h, were treated with highly purified, TLR4 specific LPS (100 ng/mL) for 15 or 30 min. Nuclear proteins from the differentially treated cells were prepared and analyzed by western blotting for nuclear p65, a subunit of NF-*κ*B. As shown in Figure 3(a), LPS treatments induced nuclear localization of p65, indicative of NF-*κ*B activation (lanes 1, 4, and 7). However, pretreatment with 2.5 *μ*g/mL or 5 *μ*g/mL of FGS did not significantly, albeit marginally by 5 *μ*g/mL of FGS, affect the nuclear localization of p65 (lanes 5–9 compared to lanes 4 and 7), suggesting that FGS may not affect NF-*κ*B activity.

Since NF-*κ*B plays a key role in regulating the expression of proinflammatory cytokines such as TNF-*α* and IL-1*β* [7] and inducing iNOS and thereby producing nitric oxide (NO) in macrophages [16], we further tested whether or not FGS affects NF-*κ*B activity by measuring LPS-induced TNF-*α* and NO production in macrophages. RAW 264.7 cells were pretreated with FGS and then treated with LPS (100 ng/mL). At 24 h after the treatment, the levels of TNF-*α* (Figure 3(b)) and NO (Figure 3(c)) were determined by semiquantitative RT-PCR and nitrite measurement, respectively. As shown in Figure 3(b), LPS treatment induced the expression of TNF-*α* (the 2th column), which was not significantly affected by FGS treatments (3th, 4th, and 5th columns). In experiment for the effect of FGS on IL-1*β* expression, we obtained similar results (data not shown). As shown in Figure 3(c), LPS treatment induced the production of a significant amount of NO (the 4th column), which was not significantly affected by FGS treatments either (the 5th and 6th columns). Together, these results suggest that FGS in our experimental conditions does not affect NF-*κ*B activity. 

### 3.3. FGS Activates Nrf2 and Induces the Expression of Nrf2-Regulated Genes

**Figure 4:**
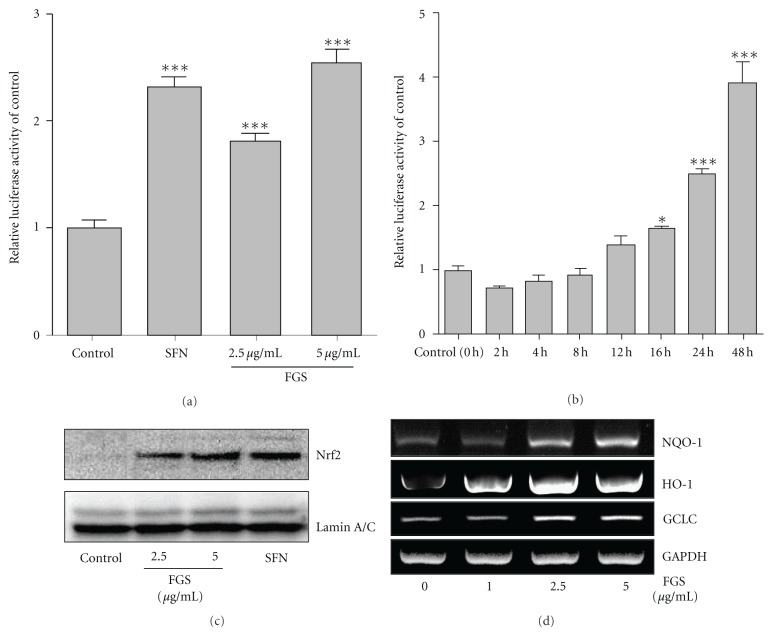
FGS activates Nrf2 and induces the expression of its dependent genes. An Nrf2 reporter cell line, derived from RAW 264.7 cells, was treated with SFN (5 *μ*M) or the indicated amounts of FGS for 16 h (a), or treated with 2.5 *μ*g/mL of FGS for various periods (b). Luciferase activity was normalized by the amount of total proteins. Data represent the mean ± SEM of triplicate settings, and experiment was repeated at least three times and shown are representatives (*P < 0.05 and ***P < 0.001, compared to control). (c) RAW 264.7 cells were treated with FGS for 16 h, and nuclear Nrf2 was measured by Western Blot analysis. (d) Expressions of Nrf2-dependent genes by FGS and internal controls were determined by semiquantitative RT-PCR.

Since our results show no significant involvement of FGS in NF-*κ*B activity, we explored another possibility that the anti-inflammatory function of FGS is mediated by activating Nrf2, an anti-inflammatory transcription factor that prevents from acute lung inflammation [13, 17, 18]. To test this, we created an Nrf2 responsive reporter cell line derived from RAW 264.7 cells. The cell line harbors a 1 kb long NQO-1 proximal promoter fused with firefly luciferase gene. As shown in Figure 4(a), treatment of the cell line with Sulforaphane, a well-documented Nrf2 activator, increased luciferase activity, suggesting the responsiveness of the reporter cell line to activated Nrf2 (columns 1 and 2). Treatment of the reporter cell line with FGS, 2.5 or 5 *μ*g/mL, for 16 h induced luciferase activity (columns 4 and 5), demonstrating that FGS activates Nrf2. To determine the efficacy of FGS in activating Nrf2, we treated the reporter cells with FGS (2.5 *μ*g/mL) for various periods. As shown in Figure 4(b), FGS treatment progressively increased luciferase activity. 

Next, we investigated whether the increased luciferase activities by FGS treatment are correlated with nuclear localization of Nrf2, indicative of activated Nrf2. After similar treatments of RAW 264.7 cells with various amounts of FGS, the nuclear proteins were extracted from the treated cells and analyzed by western blotting for nuclear Nrf2. As shown in Figure 4(c), Nrf2 was detected in the nucleus after FGS treatment. To test whether FGS treatment also induces Nrf2-regulated gene expression, we performed similar experiment, extracted total RNA, and determined the expression of NQO-1, GCLC, and HO-1 by semiquantitative RT-PCR analysis. As shown in Figure 4(d), FGS treatment induced the expression of Nrf2-regulated genes. Taken together, these results show that FGS activated Nrf2, resulting in Nrf2-dependent gene expression.

**Figure 5:**
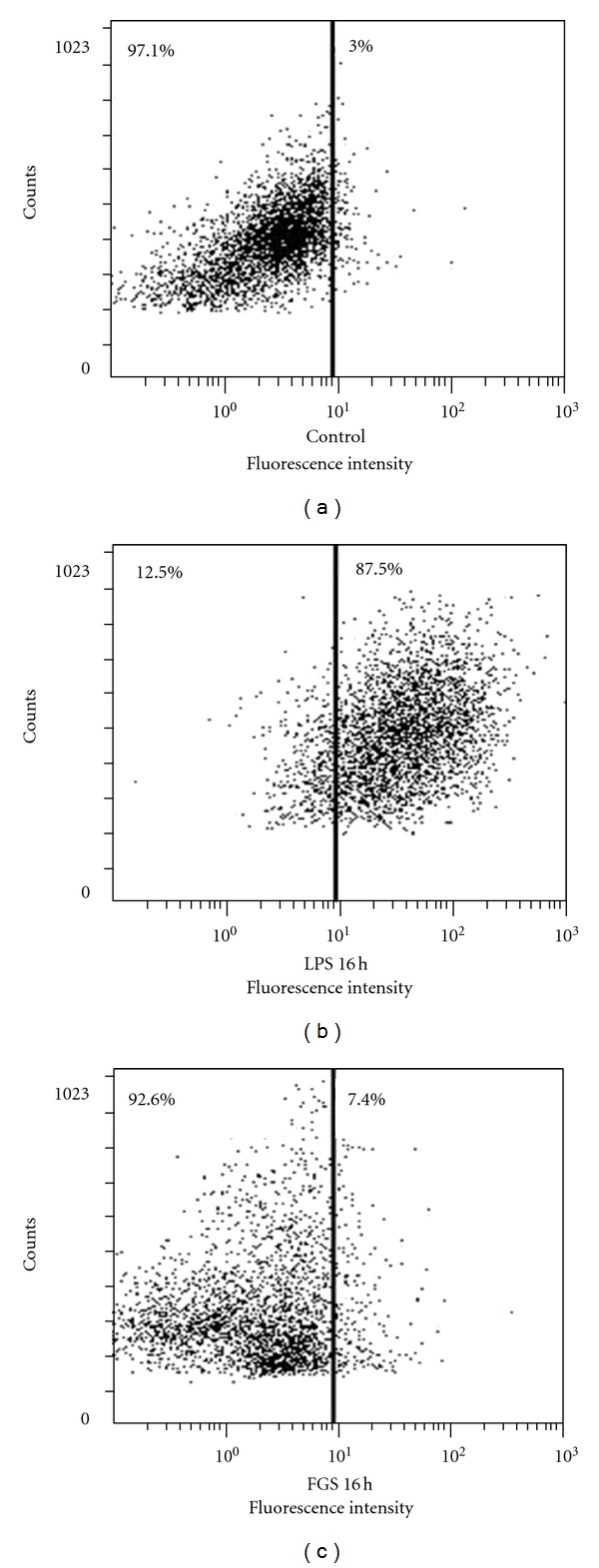
FGS does not significantly generate intracellular ROS generation. RAW 264.7 cells were treated with LPS (100 ng/mL) or FGS (25 *μ*g/mL) for 16 h, and ROSs were measured by flow cytometric analysis. Experiment was repeated three times, showing no significant production of ROS by FGS compared to controls.

Finally, given that reactive oxygen species (ROS) exacerbates inflammation and activates Nrf2 [19], we sought to exclude the possibility that FGS or other impurity, if any, in FGS induces ROS production, resulting in Nrf2 activation. To this end, RAW 264.7 cells were treated with FGS (25 *μ*g/mL), 10 times higher than the amount used in this study, for 16 h, and stained with carboxy-H_2_DCFDA prior to flow cytometric analysis. As shown in Figure 5, while LPS treatment induced ROS production, FGS did not significantly elicit ROS production. In a similar experiment in which the cells were treated with 5 *μ*g/mL of FGS, the level of ROS was virtually same as that of control (data not shown). Together, these results suggest that the anti-inflammatory effect of FGS is mediated, at least in part, by activating Nrf2 without mediation of ROS.

### 3.4. FGS Enhances the Expression of Nrf2-Dependent Genes but Suppresses That of NF-*κ*B-Dependent Genes in Inflamed Mouse Lungs

**Figure 6:**
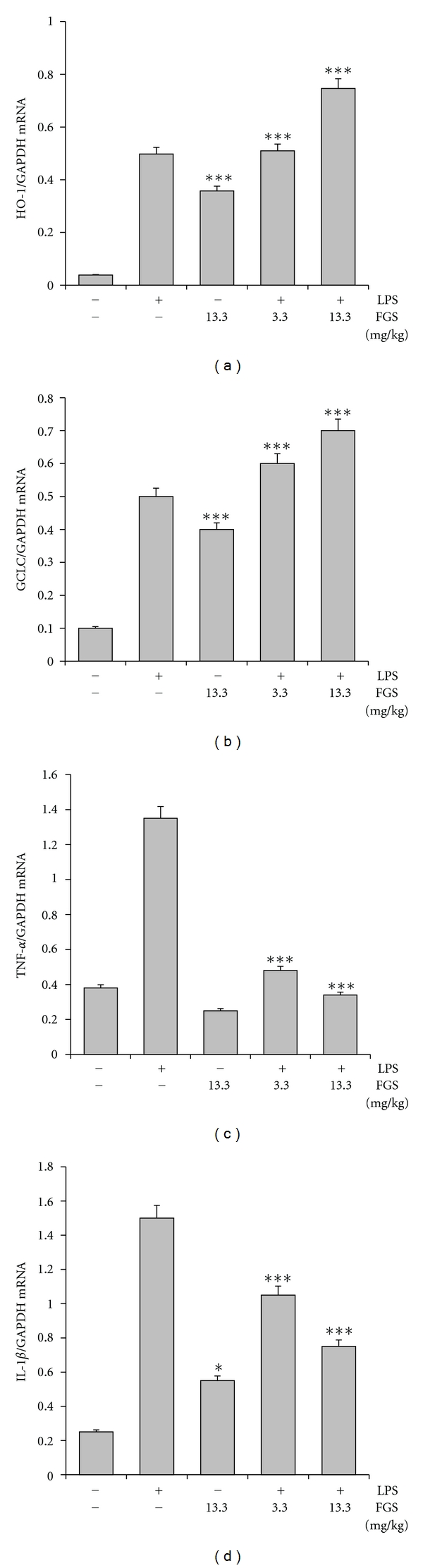
FGS enhances the expression of Nrf2-dependent genes but suppresses that of NF-*κ*B-dependent genes in mouse lungs. Mice (*n* = 5 in each group), fed with either PBS or the indicated amounts of FGS, received an intranasal LPS (10 mg/kg of a mouse) or sham. Expressions of HO-1 (a), GCLC (b), TNF-*α* (c), and IL-1*β* (d) in the lungs were measured by semiquantitative RT-PCR. The intensity of each PCR band was measured by densitometer and software (ImageJ), and relative expression of each gene was normalized against that of GAPDH. Data represent the mean ± SEM. FGS induced the expression of HO-1 (a) and GCLC (b) in mouse lungs, and ***P < 0.001, compared to the untreated control. LPS instillation to mouse lungs induced the expression of TNF-*α* (the 2nd column in (c)) and IL-1*β* (the 2nd column in (d)), which were reduced by FGS (***P < 0.001, compared to LPS-treated control). FGS treatment alone induced the expression of IL-1*β* (d); *P < 0.05, compared to the untreated control).

To test whether the suppressive effect of FGS on lung inflammation is associated with Nrf2 activation, we performed the similar experiment described in Figure 1, and determined the expression levels of Nrf2- and NF-*κ*B-dependent genes in inflamed lungs. Mice (*n* = 5 in each experimental group) were fed with either ddH_2_O or two different doses of FGS (3.3 and 13.3 mg/kg of a mouse) for 15 days prior to administration of intranasal LPS (10 mg/kg of a mouse) or sham. At 24 h after the treatments, total RNA extracted from the lungs was analyzed by semiquantitative RT-PCR for the expression of Nrf2-dependnet (Figures 6(a) and 6(b)) and NF-*κ*B-dependent genes (Figures 6(c) and 6(d)). As shown in Figure 6(a), FGS treatment induced the expression of HO-1 expression (the 2nd column), which is likely the result of ROS produced during inflammatory reaction in the lung. FGS treatment induced HO-1 expression (the 3rd column), which was further increased in inflamed lung (4th and 5th columns). Similar increases of the expression of GCLC-1 were observed in inflamed lung (3rd, 4th, and 5th columns in Figure 6(b)). On the other hand, consistent with the results in Figure 1, LPS instillation substantially induced the expression of proinflammatory cytokines such as TNF-*α* (the 2nd column in Figure 6(c)) and IL-1*β* (the 2nd column in Figure 6(c)), which were, however, suppressed by FGS in a dose-dependent manner (4th and 5th columns in Figures 6(c) and 6(d)). These results suggest that FGS induces the expression of Nrf2-dependent genes and suppresses that of NF-*κ*B-dependent genes in the inflamed lungs.

## 4. Discussion

Since A.D. 2 in China and eastern Asian countries, the fruit hull of *G. sinensis *(FGS) has been prescribed for the treatment of various respiratory symptoms such as cough, wheezing, and other respiratory conditions with abscess [20, 21]. However, it remains unknown how FGS exerts its effect. Given these recorded effects of FGS on respiratory diseases, we postulate and test whether FGS suppresses inflammation by regulating key inflammatory transcription factors. We demonstrated the suppressive effect of FGS on acute neutrophilic lung inflammation in ALI/ARDS in a mouse model and provided evidence that FGS executes its anti-inflammatory function via activation of an anti-inflammatory factor Nrf2. Our results suggest that the therapeutic effect of FGS is attributable to suppression of inflammation, which is likely mediated by, at least in part, activating Nrf2 but not suppressing proinflammatory factor NF-*κ*B. To our best knowledge, this is the first experimental evidence showing that FGS is effective in regulating acute neutrophilic lung inflammation and implicated in Nrf2 activation.

FGS is composed of numerous constituents including tannins, resins, wax alcohols, beta-sitosterol, stigmasterol, galactose, mannose, and saponins such as gledinin, gleditsia saponins [20, 22–25]. Although our results suggested anti-inflammatory effect of FGS, it is also highly likely that some of the constituents have other effects. For example, as shown in Figure 5, FGS treatment increased the size of the treated cells compared to controls, as the size scattering became diffused in FGS-treated cells. In addition, FGS treatment alone increased the production of IL-1*β* in inflamed lung (Figure 6(d)), although LPS induced, robust inflammation in the lung was reduced by FGS. Nevertheless, our results strongly suggest that the collective effect of FGS was the suppression of inflammation. A recent study reported that FGS has a bacteriostatic effect on *Staphylococcus aureus, Bacillus subtilis,* and *Escherichia coli* (*E. coli*) [26], the effect of which was attributed to gleditsia saponin [27]. Since one of the major causes of ALI/ARDS is septic infection by bacteria and our results show that FGS suppressed LPS-induced neutrophilic lung inflammation, a key feature of ALI/ARDS, it is likely that FGS is also effective in treating ALI/ARDS caused by Gram-negative bacterial sepsis. 

NF-*κ*B is a major transcription factor that governs the expression of proinflammatory cytokines and chemokines [28]. The roles of NF-*κ*B in severe respiratory diseases such as septic ALI and ARDS have been well documented, and thus numerous therapeutic strategies to cure ALI/ARDS have largely hinged on curbing NF-*κ*B activity. However, those strategies have shown no significant clinical effects [29, 30]. Rather, emerging evidence shows that Nrf2 plays a key role in protecting from various respiratory diseases such as acute lung inflammation, smoke-induced emphysema, and asthma [13, 17, 31, 32]. Nrf2, a member of the Cap'n'collar basic region leucine zipper (CNC-bZIP) transcription factor family, was originally known to activate cellular protective pathways against oxidative injury [33]. Although ubiquitously expressed, this nuclear factor is located abundantly in tissues where detoxifying reactions routinely occur, such as intestine, kidney, and lung [34]. Thus, regulation of Nrf2 can be an effective therapeutic target in modulating inflammatory lung diseases.

Our results show that FGS had a suppressive effect on lung inflammation in an acute lung injury mouse model. RT-PCR analyses of lung sections indicate that FGS enhanced the expression of Nrf2 dependent genes but suppressed that of NF-*κ*B-dependent genes, suggesting that FGS suppresses acute lung inflammation by both activating Nrf2 and suppressing NF-*κ*B. However, the data obtained from molecular biologic analyses of macrophages suggest differently: FGS only activates Nrf2 without interfering the activity of NF-*κ*B. Our study has a limitation in addressing Nrf2 activation in a cell type specific manner, especially in the lung. Thus, it is conceivable that FGS selectively suppressed the activity of NF-*κ*B in other parenchymal cells such as neutrophils in the lung, but not in macrophages. Since Nrf2 activation in macrophage cannot directly turn off the expression of NF-*κ*B-dependent genes in the same cell [35], it is unlikely that Nrf2 activated by FGS in a particular parenchymal cell type was directly involved in suppressing the expression of NF-*κ*B-dependent genes in the same cell type in the lung. Rather, it is possible that Nrf2 induced the expression of genes that could null the proinflammatory responses in the context of the inflammatory milieu. For instance, inflammatory response involves reactive oxygen species (ROS) that exacerbate inflammation [36]. At the same time, ROSs are a potent activator of Nrf2 that enhances the expression of ROS scavengers such as NQO-1 and GCLC, resulting in reduced expression of proinflammatory cytokines such as TNF-*α* and IL-1*β*. These possibilities warrant further studies. Nevertheless, our results suggest that FGS contains lead compounds that are effective in treating ALI/ARDS, suggesting that FGS can be an option for the treatment of ALI/ARDS patients.

 Oxidative stress culminated during inflammatory responses activates Nrf2 because macrophages and neutrophils, key effector cells in regulating inflammatory responses, produce ROS to remove pathogens. ROS, however, causes damage to nearby cells, which in turn activates Nrf2 and induces Nrf2-dependent gene expression in inflicted cells as a protective measure [37]. Although an effective arsenal for removing pathogens, ROS can cause collateral tissue damage. Therefore, therapeutics that activate Nrf2 without generating ROS would be desirable to suppress inflammation without unnecessary tissue damage. Our results show that FGS activated Nrf2 without significantly generating ROS and induced Nrf2-dependent protective genes, suggesting that FGS can mediate anti-inflammatory functions with no significant side effects.

## 5. Conclusions

Here, we provide, to our best knowledge, the first experimental evidence suggesting that FGS pretreatment protected from acute neutrophilic lung inflammation, the hallmark of ALR/ARDS, and that FGS activated an anti-inflammatory factor Nrf2 without affecting NF-*κ*B activity. These results suggest that the therapeutic effect of FGS in traditional usage for various respiratory diseases stems from suppression of inflammation, which is associated with activation of anti-inflammatory transcription factor. Our results also raise the possibility that FGS can be used as an option for the treatment of ALI/ARDS.
